# Outpatient management of patients presenting with venous thromboembolism: Retrospective cohort study at 11 community hospitals

**DOI:** 10.1007/s11239-020-02328-9

**Published:** 2020-11-07

**Authors:** Rasha Khatib, Kara Nitti, Marc McDowell, Rick Szymialis, Chris Blair, Nicole Glowacki, William Rhoades

**Affiliations:** 1 Advocate Aurora Research Institute, Advocate Health Care, Downers Grove, IL USA; 2 Advocate Christ Medical Center, Oak Lawn, IL USA; 3 Bristol-Myers Squibb, Princeton, NJ USA; 4 Advocate Lutheran General Hospital, Park Ridge, IL USA

**Keywords:** Home management, Venous thromboembolism, Clinical practice guidelines, Low risk, PESI

## Abstract

A gap exists between clinical practice guidelines and real-world practice. We aim to investigate hospital admissions among patients presenting to emergency departments of 11 hospitals with venous thromboembolism (VTE). Eligible patients’ first emergency department VTE visit were retrospectively collected between 2013 and 2018 from electronic medical records (EMR). Patients were categorized at low risk of VTE complications if they were diagnosed with deep vein thrombosis (DVT) of the leg or if they were diagnosed with pulmonary embolism (PE) and had a PE score index < 85. Multivariable logistic regression models were constructed to measure the adjusted odds ratios (OR) and 95% confidence intervals (CI) of hospital admissions before and after clinical practice guidelines were updated to recommend outpatient management of DVT and PE with low risk of complications. A total of 13,677 patients were included in the analysis, of which 55% were diagnosed with DVT. Mean age was 65  ±  17 years, 54% were females, and 62% were Caucasian. Overall, 9281 patients were categorized at low risk VTE complications, of whom 77% were admitted for in-hospital management. The rate of in-hospital management declined from 81% in 2013 to 73% in 2018. Patients visiting emergency departments between 2016 and 2018 (post-guidelines) were equally likely to be admitted compared to patients visiting the emergency departments between 2013 and 2015 (pre-guidelines; OR = 0.99; 95% CI: 0.88, 1.11). Results from this real-world study indicate that most low-risk VTE patients are admitted for in-hospital management, despite recommendations in clinical practice guidelines.

## Highlights

- Most low risk VTE patients (67%) are admitted for in-hospital management despite evidence for safety and efficacy of outpatient management.
- Adopting clinical guidelines and integrating evidence on new and existing treatment advances remains a challenge for clinical practice.

## Introduction

Venous thromboembolism (VTE), comprising pulmonary embolism (PE) and deep vein thrombosis (DVT), occurs for the first time in 100 per 100,000 persons each year in the Unites States [1]. VTE is traditionally managed with vitamin K antagonists (VKA) and more recently with oral direct factor Xa inhibitors (DOAC). The goal of treatment is to prevent the extension of thrombus, PE, and to relieve symptoms in the short term while preventing recurrent events in the long-term [2, 3]. The prognosis of patients diagnosed with VTE is related to initial hemodynamic status. For example, the presence of systemic hypotension, cardiogenic shock and severe dyspnea in PE results in poor prognosis and high-risk of complications, including a 30 day mortality rate of 15% [4]. Patients at high-risk of complications represent 6% of patients with DVT and 10% of patients with PE [5].

There is evidence from randomized controlled trials that patients diagnosed with PE and DVT at low risk of complications may be treated at home (an outpatient setting) without the need for an inpatient admission [6, 7]. This evidence has promoted changes in clinical practice guidelines recommending outpatient treatment or early discharge, over standard discharge, in VTE patients with a low-risk of complications and whose home circumstances are adequate [2, 8].

Integration of evidence in clinical practice is slow and maybe due to provider level barriers such as lack of knowledge, patient level barriers such as lack of medication adherence, or environmental factors such as lack of time or resources [9]. Observational studies conducted in the United States report that only 1–8% of patients diagnosed with PE are discharged for outpatient management, as recommended in clinical practice guidelines [10–12]. Information on outpatient management of patients with DVT is limited. Further, these studies do not stratify by risk of VTE complications and were all conducted prior to 2015, before DOACs were indicated for VTE treatment and routinely used in clinical practice.

We aim to assess to what extent clinical practice guideline recommendations are adhered to in terms of discharging patients with low risk of VTE complications presenting to emergency departments of community hospitals for outpatient management rather than admitting them for hospital management. We also explore rates of admission over time, among those with low risk of VTE complications and examine possible predictors associated with admitting patients with low risk of VTE complications for inpatient management.

## Methods

### Study design and data sources

This is a retrospective hospital-based cohort study using electronic medical record (EMR) data from 11 Advocate Aurora Health (AAH), Illinois hospitals. EMR data across all hospitals is collected through Cerner and is available for research through AAH Electronic Data Warehouse (EDW). During the study period no policy or guideline for the treatment of venous thromboembolism were utilized within the healthcare system. Anticoagulant choice and patient management and disposition were at the discretion of individual healthcare providers. Eligible patients’ first VTE visit (index visit) were retrospectively collected from January 1st, 2013 to December 31st, 2018. Patient charts were reviewed for three months past the index visit to examine adverse events. Data extracted included patient demographics, clinical characteristics, diagnoses, treatment in the emergency department, treatment during hospital stay, length of stay, discharge disposition, and readmissions due to adverse events. The study was approved by Advocate Health Care Institutional Review Board.

### Participants

Patients were included if they were ages 18 years or older and presented to one of the 11 emergency departments with a diagnosis of VTE. Patients’ first visit was identified as the index visit. Diagnoses were identified using the International Classification of Diseases, Ninth Revision (ICD-09) codes and the International Classification of Diseases, Tenth Revision (ICD-10) codes derived from a previously published systematic review [13].

Clinical practice guidelines recommend home management of patients with VTE at low risk of complications; we therefore stratified our patient population by risk [2, 8]. Patients were categorized as having low risk of VTE complications if they were diagnosed with DVT of the leg [2] or if they were diagnosed with PE and had aPE score index (PESI) ≤ 85 (Table 4 in Appendix 1) [14].

Patients who were pregnant during the index visit were excluded from the analysis. To account for possible inaccuracies in diagnosis codes in EMR data, patients who were not administered a nonprophylactic dose of anticoagulant during the index visit or at discharge were excluded. Further, patients who received an anticoagulant at a prophylactic dose were excluded. A prophylactic dose was defined as subcutaneous unfractionated heparin and/or 30 mg and 40 mg of enoxaparin. Patients who expired during the index visit and patients with missing information to determine VTE severity (PESI score items/location of DVT), were excluded from the analysis.

### Primary predictor

Patients were grouped by period of admission into pre-clinical guideline emergency department visits and post- clinical guideline emergency department visits. Pre- and post- periods refer to visits between 2013 and 2015, before CHEST guidelines on antithrombotic therapy for VTE disease were issued, and visits between 2016 and 2018, after CHEST guidelines were issued. These guidelines recommend outpatient management of patients diagnosed with a PE at low risk of complications [8].Guidelines recommending outpatient management of DVT were issued in 2012, which precedes this study timeline [2].

### Outcomes

The primary outcome is admission among patients diagnosed with VTE at low risk of complications. The secondary outcome is hospital length of stay among low risk patients who were admitted for VTE management.

### Variables

Comorbidities and risk factors were identified from the EMR using a list of ICD-9 and 10 codes developed and reviewed by a clinician for the purposes of this study (Fig. 4 in Appendix 2). Stroke was defined as ischemic stroke, hemorrhagic stroke, or history of stroke. Chronic lung disease was defined as having chronic obstructive pulmonary disease, asthma, or pulmonary fibrosis. Cancer was defined as having an active malignancy or a malignancy in remission. Surgery was defined as requiring a surgery with general or epidural anesthesia within three days prior to the index visit. Bleeding during the hospital stay and at 3 months was determined based on ICD codes reported as diagnoses in the EMR. Recurrent VTE events were defined as emergency department or hospital admission due to VTE, occurring up to 3 months after the index visit. The same definition was used for index VTE and recurrent VTE.

Pharmacist presence was identified based on a time proxy: if a patient presented to the emergency department during pharmacy off hours (1:30 AM-5:59 AM), it was assumed there was no pharmacist present. Alternatively, if a patient presented to the emergency department during pharmacy hours (6:00 AM-1:29 AM), it was assumed a pharmacist was present.

### Statistical analysis

Patient demographics and clinical characteristics are presented with means and standard deviations (SD) or medians and interquartile ranges (IQR) as applicable for continuous variables, and as proportions and absolute numbers for categorical variables. Multivariable logistic regression models were created to examine the association of guideline issuance at the time of visit to VTE admission and to identify additional possible predictors to admission. Model results are presented as adjusted odds ratios (OR) and 95% confidence intervals (CI). The following models were adjusted for index year, age, sex, race, ethnicity, insurance, VTE type, obesity, hypertension diagnosis, heart failure, cancer, stroke, chronic kidney disease, anticoagulant type, presence of a pharmacist, and teaching hospital.

## Results

Over the 6 year study period, 2,193,965 emergency department visits were identified from the EMR, of which 20,027 unique patients were diagnosed with VTE. After applying the inclusion criteria, a total of 13,677 patients were included in the analysis (Table 5 in Appendix 3). Patient characteristics are presented by VTE management in Table 1. The mean (SD) age was 64.8  ±  17.5 years and 53.7% were female. Over half of the patients were Caucasian (62.3%), and most had commercial (44.4%) or Medicare (44.7%) insurance. DOAC was administered to 29.6% of patients, and a pharmacist was present during 91.3% of patient visits. Overall, 81.6% of patients were admitted for in-hospital management.

**Table 1:** Demographic and clinical characteristics by VTE management

	Discharged for outpatient management	Admitted for in hospital management	Total
Overall	2,517 (18.4%)	11,160 (81.6%)	13,677 (100%)
Mean age + SD, years	58.0 ± 17.5	66.4 ± 17.1	64.8 ± 17.5
> 65 years	886 (35.2%)	6,250 (56.0%)	7,136 (52.2%)
Sex, female	1,284 (51.0%)	6,065 (54.4%)	7,349 (53.7%)
Race			
Caucasian	1,642 (65.2%)	6,875 (61.6%)	8,517 (62.3%)
African American	673 (26.7%)	3,573 (32.0%)	4,246 (31.0%)
Asian or Other	93 (3.7%)	336 (3.0%)	429 (3.1%)
Null, Declined, Missing	108 (4.3%)	376 (3.4%)	485 (3.6%)
Ethnicity, Latino	217 (8.6%)	725 (6.5%)	942 (6.9%)
Insurance			
Commercial	1,541 (61.2%)	4,534 (40.6%)	6,075 (44.4%)
Medicare	693 (27.5%)	5,417 (48.5%)	6,110 (44.7%)
Medicaid	228 (9.1%)	978 (8.8%)	1,206 (8.8%)
No coverage	55 (2.2%)	231 (2.1%)	286 (2.1%)
Comorbidities			
Hypertension	989 (39.3%)	7,608 (68.2%)	8,597 (62.9)
Heart Failure	106 (4.2%)	2,422 (21.7%)	2,528 (18.5)
Cancer or history of cancer	205 (8.1%)	1,998 (17.9%)	2,203 (16.1)
Chronic lung disease	82 (3.3%)	1,145 (10.3%)	1,227 (9.0)
Chronic kidney disease	55 (2.2%)	741 (6.6%)	796 (5.8)
Anticoagulant type			
DOAC ± PAC	985 (39.6%)	2,901 (27.2%)	3,886 (29.6)
VKA ± PAC	1,505 (60.4%)	7,752 (72.8%)	9,257 (70.4)
Pharmacist present	2,367 (94.0%)	10,120 (90.7%)	12,487 (91.3)

*VTE* venous thromboembolism, *VKA* vitamin k antagonist, *DOAC* direct-acting oral anticoagulant, *PAC* premature atrial complexes, *SD* standard deviation

A total of 6166 (45.1%) patients with VTE were diagnosed as PE with a mean PESI score of 87.5  +  29.8, (49.7% had a PESI score of < 85, indicating a low risk of VTE complications). Table 4 in Appendix 1 presents additional details on calculating PESI. A total of 7511 (54.9%) patients with VTE were diagnosed as DVT. Most patients were diagnosed with DVT of the leg (82.8%), indicating low risk of VTE complications. Among all patients with VTE, 67.9% (n = 9281) were categorized as having a low risk of VTE complications (Table 2).

**Table 2:** Risk of complications among PE, DVT, and overall VTE diagnoses by VTE management

	Total (N = 13,677)
PE	6,166 (45.1)
* Mean PESI Score* + *SD*	*87.5* ± *29.8*
* PESI score* < *85, N(%)*	*3,066 (49.7)*
DVT	7,511 (54.9)
* Lower extremity DVT of the leg, N(%)*	*6,215 (82.8)*
All VTE	13,677 (100%)
* Low risk of VTE complications*^*a*^*, N(%)*	*9,281 (67.9%)*

^a^Low risk of VTE complications referred to DVT of the leg among patients diagnosed with DVT and PESI < 85 among patients diagnosed with *VTE* venous thromboembolism, *PE* pulmonary embolism, *DVT* deep vein thrombosis, *PESI* pulmonary embolism severity score, *SD* standard deviation

**Fig. 1:**
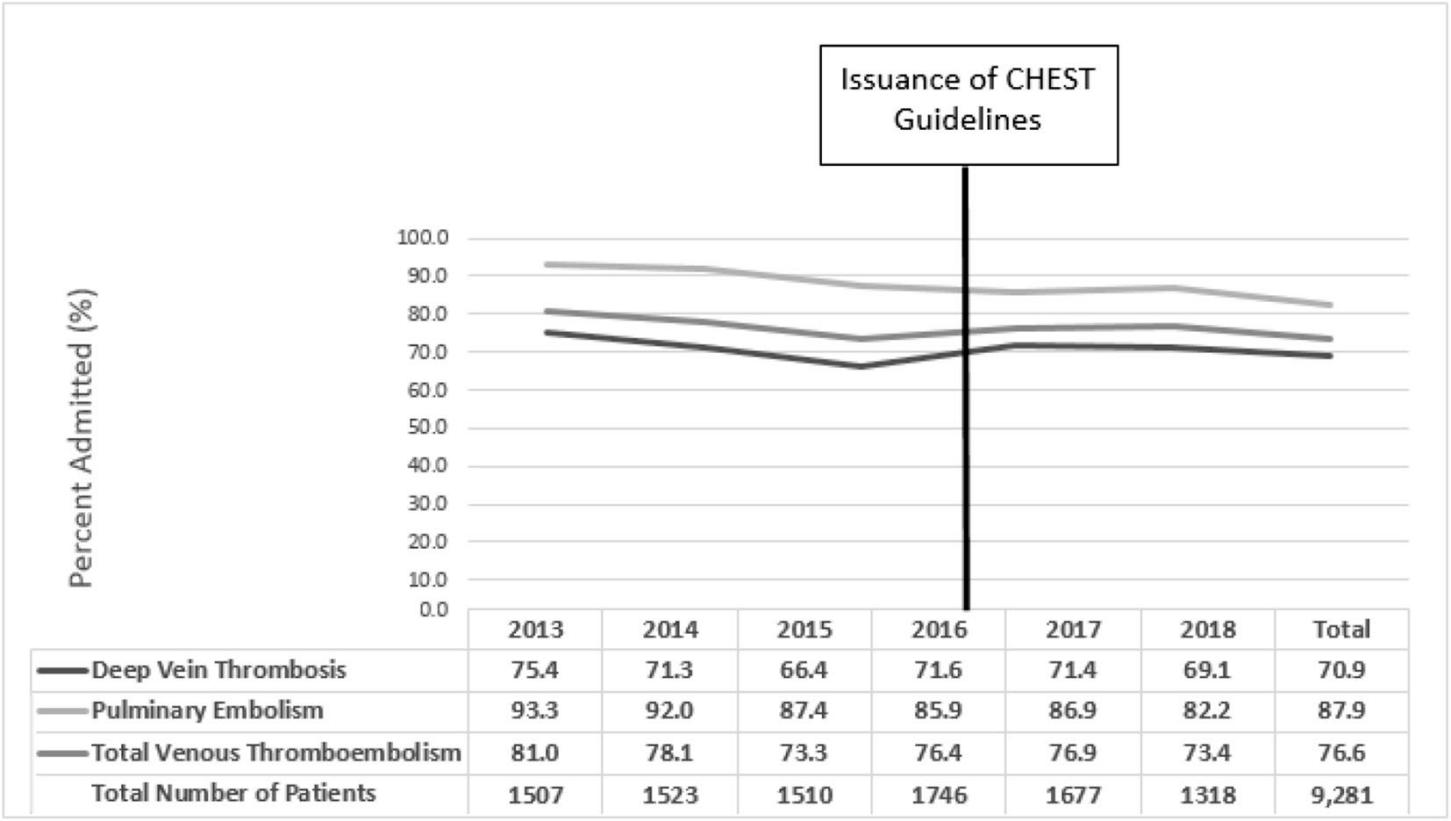
Admission rate among low risk by VTE type. *VTE* venous thromboembolism

Figure 1 presents the proportion of patients admitted for in-hospital management among those with low risk of VTE complications (n = 9281) by year and type of VTE (i.e. PE or DVT). Overall, admissions among patients with low risk of VTE complications dropped over the 6 year study period from 81.0% to 73.4% (*p* < 0.01). Overall, 76.6% of patients with a low risk of complications were admitted. However, the cumulative proportion of VTE admissions was similar before (77.4%) and after (75.7%) the issuance of 2016 CHEST guidelines (*p* = 0.05). Differences were also not statistically significantly different by type of VTE.

**Fig. 2:**
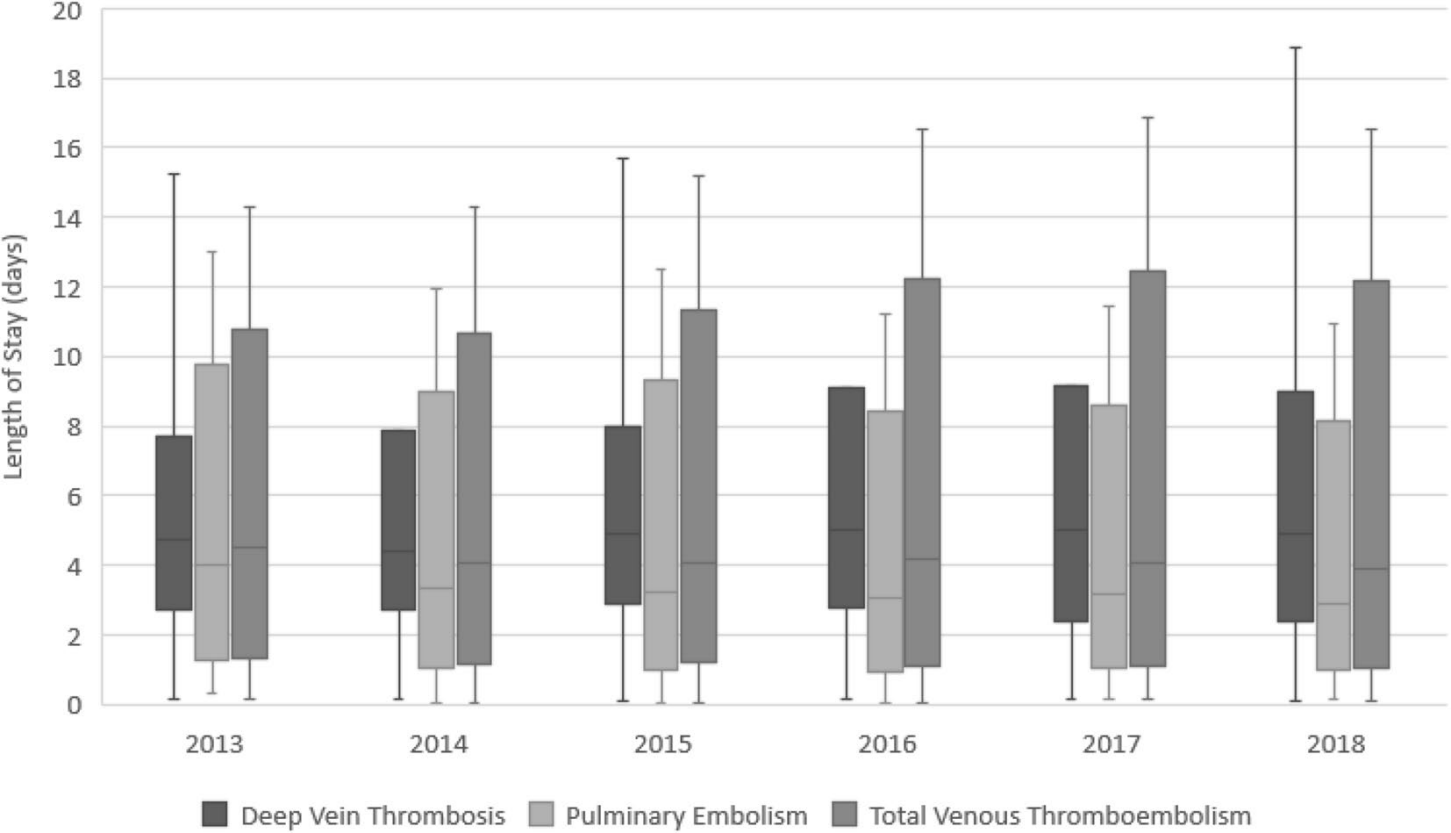
Median length of stay of admitted, low risk patients by VTE Type (Excluding Outliers). *VTE* venous thromboembolism

The median length of stay among the 7105 patients who were at low risk of VTE complications and who were admitted are presented in Fig. 2, by year. Median hospital length of stay was similar over the 6 year study period (4.5 days [IQR: 2.5–7.2] in 2013 and 3.9 days [IQR: 2.0–7.8]; *p* = 0.08) in 2018. Median length of stay was also similar before and after issuance of CHEST guidelines (4.2 days [IQR: 2.4–7.2] before and 4.0 days [IQR: 2.1–7.9] after, *p* = 0.69). Table 6 in Appendix 4 presents median (IQR) length of stay by year and VTE diagnosis, in tabular form.

**Fig. 3:**
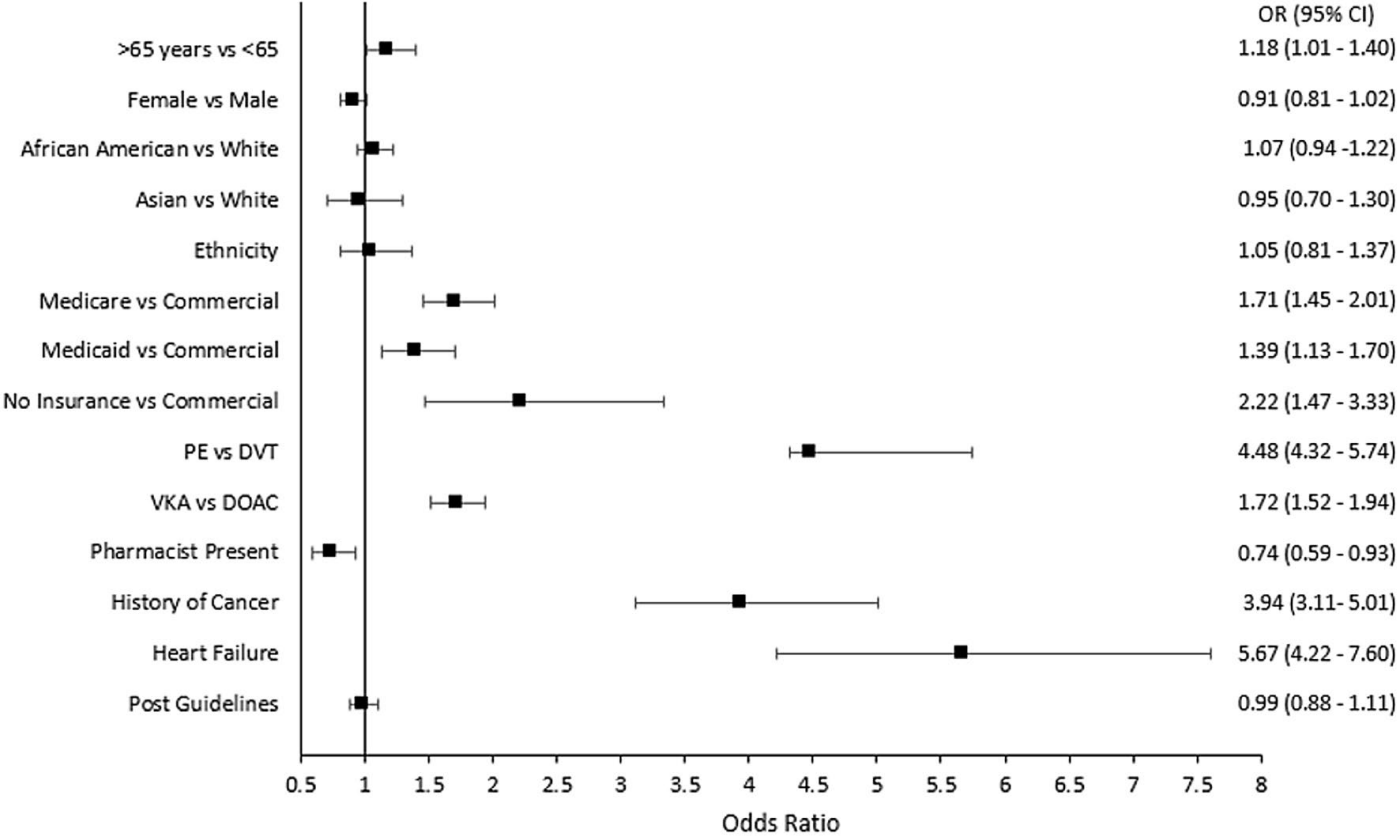
Multivariate analysis of admission rates among low risk VTE patients. Adjusted for: admit year, age, sex, race, ethnicity, insurance, venous thromboembolism type, obesity, hypertension diagnosis, heart failure, cancer, stroke, chronic kidney disease, anticoagulant type, presence of a pharmacist, and teaching hospital. *VTE*: venous thromboembolism, *PE* : pulmonary embolism, *DVT* : deep vein thrombosis, *VKA*: vitamin k antagonist, * DOAC*: direct-acting oral anticoagulant

**Fig. 4:**
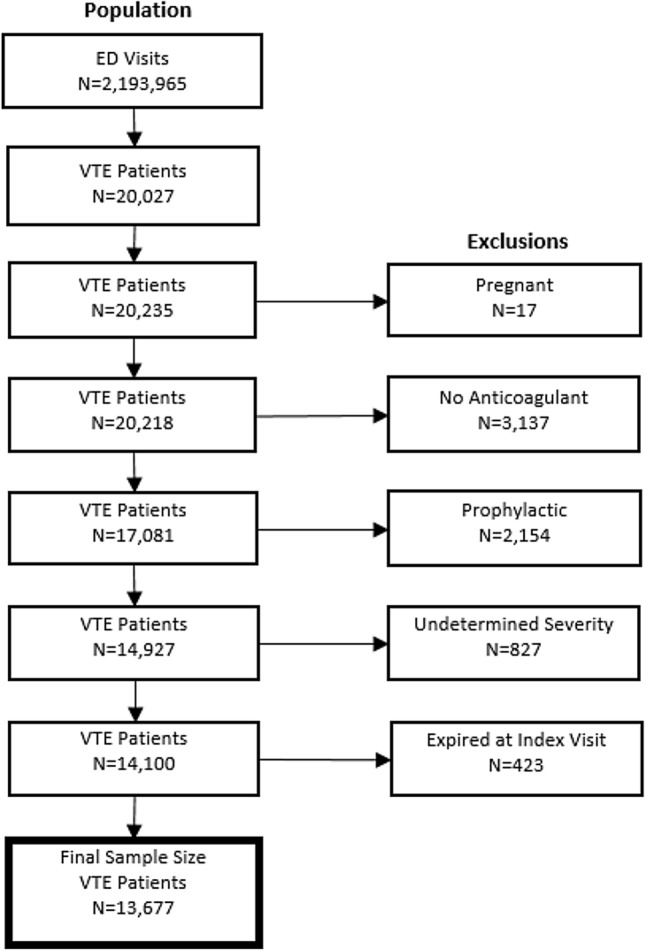
Patient Flow

Figure 3 presents possible predictors of admission among patients with low risk of VTE complications. After adjusting for several potential confounders, including comorbidities, patients were more likely to be admitted if they had Medicare (OR 1.71, 95% CI 1.45–2.01), Medicaid (OR 1.39, 95% CI 1.13–1.70), or if they were uninsured (OR 2.22, 95% CI 1.47–3.33) compared to patients with commercial insurance. Patients with PE were four times more likely to be admitted compared to patients with DVT (OR 4.48, 95% CI 4.32–5.74). Patients were 1.72 times more likely to be admitted if they were administered VKA compared to DOAC (95% CI 1.52–1.94) and less likely to be admitted if a pharmacist was present when they presented to the emergency department (OR 0.74, 95% CI 0.59–0.93). Age, sex, race, ethnicity, and timing of visit (pre- versus post- CHEST guidelines) were not associated with type of VTE management.

Three-month adverse events are reported in Table 3. Mortality at three months was higher among patients who were previously admitted compared to those who were discharged from the emergency department. Recurrent VTE and bleeding events were not statistically significantly different between the two groups.

**Table 3:** Three-month outcomes among low risk VTE patients N = 9,281 (%)

Clinical characteristics	Discharged from ED (N = 2,176)	Admitted (N = 7,105)	%Difference (95%CI)
Readmitted	546 (25.1)	2,306 (32.5)	7.36 (5.24, 9.49)
Presented to ED	1 (0.1)	4 (0.1)	0.01 (−0.10, 0.12)
Mortality	8 (0.4)	191 (2.7)	2.32 (0.23, 1.87)
Recurrent VTE	94 (4.3)	199 (2.8)	−1.53 (−2.47, −0.60)
Bleeding	29 (1.3)	130 (1.8)	0.05 (−0.08, 1.07)

Results are limited to patients who were alive at the end of their index visit

*VTE* venous thromboembolism, *ED* emergency department

## Discussion

This is a retrospective cohort study of emergency departments from 11 community hospitals in a large integrated healthcare system in the United States. Over the 6 year study period, 81.6% of patients diagnosed with VTE were admitted for inpatient management. Specific to patients categorized at low risk of VTE complications, 76.6% were admitted. We explored trends of in-hospital admissions over time. The proportion of low risk patients admitted was 81% in 2013 and decreased to 73.4% in 2018.

Clinical practice guidelines have recommended outpatient management of patients with DVT and low risk of complications since 2012 [6]. In 2016, these guidelines were updated to also include outpatient management of patients with PE and low risk of complications [6, 7]. Our results indicate minimal change in rates of outpatient management of VTE after guidelines were updated. Several studies have investigated integration of evidence and clinical guideline recommendations into clinical practice and results consistently indicate minimal and slow uptake of the evidence [15]. Multiple types of barriers to practice change have been reported, including lack of awareness or familiarity with current recommendations, lack of agreement with the recommendations, lack of self-empowerment to make practice changes, inertia, and external barriers to practice change [9]. Understanding the extent of implementation of evidence in clinical practice is critical for improving patient safety and health outcomes. This information should be incorporated to design interventions and policies to encourage use of effective treatments and use limited health care budgets effectively [15].

There are several explanations for the observed high rates of in-hospital management for patients with VTE, despite what is recommended in clinical practice guidelines. First, guidelines specify using PESI or other validated prediction tools to identify patients at low risk of complications. Incorporating such tools in emergency department clinical protocols may help providers identify patients at low risk who could be managed in an outpatient setting. Second, clinical practice guidelines specify outpatient management to patients at low risk of complications and “whose home circumstances are adequate” [8]. We attempted to adjust for insurance status as a proxy for patient circumstances and did show that patients with commercial insurance were less likely to be admitted. Social needs, such as lack of shelter or lack of social support at home, were not explored in our analysis but may explain why some patients were at low risk of complications but were still admitted. Third, guidelines recommend that a robust outpatient follow-up plan be in place if patients are to be managed in an outpatient setting. Further, exploration is required to assess if these plans exist and if they are feasible within this healthcare system. Fourth, providers may be less comfortable with discharging patients at low risk of complications if they have other comorbidities. In fact, our data indicates that heart failure and cancer comorbidities were strong predictors for in-patient admission, despite an overall low risk of VTE complications. Fifth, presence of a pharmacist appears to be associated with greater likelihood of discharging patients for outpatient management, in alignment with clinical practice guidelines. This may be due to pharmacists’ involvement and knowledge of existing treatment plans in outpatient settings [16]. Sixth, our results indicate that patients administered DOAC were more likely to be discharged for outpatient management. As clinical practice evolves, the adoption of DOACs will become more prevalent. The ease of administration and the lack of need for bridging oral therapy with parenteral anticoagulation (as with warfarin, which is recommended until desired international normalized ratio (INR) is achieved) affords otherwise healthy patients a way to avoid these risks. These possible explanations should be addressed in attempts to overcome barriers to implementing outpatient VTE management recommendations in large healthcare systems.

Our results demonstrate there remains a large opportunity to treat low-risk VTE in the outpatient setting. Avoiding an admission for this subset of patients would offer a variety of benefits to providers, institutions, and patients, including nosocomial infections, iatrogenic medication errors, additional resource utilization, and higher healthcare costs.

Observational studies investigating outpatient management of VTE and results are consistent with our findings indicating that in the clinical setting most VTE patients are managed in the hospital, despite available evidence and recommendation in clinical practice guidelines regarding the safety and efficacy of outpatient management. Unlike previous studies, we were able to stratify patients by VTE risk, we included patients with PE and DVT, and we were able to explore temporal trends covering a period before and after clinical practice guidelines were updated to include recommendations for outpatient management of DVT and PE [10–12]. However, our study has limitations. Although we categorized VTE patients by risk of complications, we may have miscategorized patients based on unmeasured comorbidities or social needs which we did not account for in our analysis. We used EMR data and were limited to data routinely collected in the clinical setting. We also were not able to validate patient outcomes including mortality. Further, by nature of using EMR data, 3-month adverse events are limited to patients who were readmitted or visited an emergency department within the same healthcare system. However, our results are consistent with findings from RCTs which indicated no difference in adverse events between hospital and outpatient management [6, 7].

In conclusion, most patients who present to the emergency department of community hospitals with VTE and who are at low risk of complications continue to be admitted for hospital management, rather than discharged for outpatient management as clinical practice guidelines recommend. Our results support the literature indicating slow integration of evidence into practice and highlight the need for education on clinical practice guidelines supporting appropriate outpatient treatment of VTE.

## Data Availability

Data queries should be addressed to Advocate Aurora Research Institute.

## Appendix 1

See Table 4

**Table 4:** Pulmonary embolism severity score among PE patients

	Discharged for outpatient management (N = 520)	Admitted for in hospital management (N = 5,646)	Total (N = 6,166)
Predictors of PESI score			
Mean age + SD, years	57.3 ± 17.2	65.0 ± 17.0	64.3 ± 17.2
Gender, male	226 (43.5%)	2,446 (43.3%)	2,672 (43.3%)
Cancer or history of cancer	74 (14.2%)	958 (17.0%)	1,032 (16.7%)
Heart failure	38 (7.3%)	1,144 (20.3%)	1,182 (19.2%)
Chronic lung disease	35 (6.7%)	674 (11.9%)	709 (11.5%)
Heart Rate1 > 110 beats/min	62 (11.9%)	1,458 (25.8%)	1,520 (24.7%)
Systolic blood pressure < 100 mmHg	10 (1.9%)	340 (6.0%)	350 (5.7%)
Respiratory rate > 30 breaths/minute	8 (1.5%)	289 (5.1%)	297 (4.8%)
Temperature < 36C	10 (1.9%)	163 (2.9%)	173 (2.8%)
Altered mental status	2 (0.4%)	98 (1.7%)	100 (1.6%)
Arterial oxygen saturation < 90%	6 (1.2%)	481 (8.5%)	487 (7.9%)

*PESI* pulmonary embolism severity score, *PE* pulmonary embolism, *SD* standard deviation

.

## Appendix 2

See Fig. 4 .

## Appendix 3

See Table 5

**Table 5:** List of ICD-9 and 10 codes: comorbidities, risk factors, and bleeding

Diagnosis	ICD-9	ICD-10
Cancer	140.0–165.9, 179.0–209.36	C00.0–C96.Z, D03.0–D45.0
Stroke	433- 4.35.9, V12.54	I63.00–I66.9, Z86.73
Heart Failure	398.91, 402, 404, 428,	I09.81, I11.0, I13.2, I50
Hypertension	401–405.99	I10–I16.9
Immobility	344.1–342.92	M62.3, G81
Altered mental status	780.97	R41.82
Asthma	493.92	J45
Chronic Kidney Disease	N18.9	N18.9
COPD	491.22	J44
In-hospital bleeding	285.1, 430–432.9, 455.2–456.2, 530.7–537.83, 562.02–562.13, 568.81–569.85, 578, 423.0x, 459.0x, 599.7x, 719.11, 784.7x, 784.8x, 786.3x	I60, I61, K64.4, K64.8, I85.0, I85.11, K22.6–K31.81, K57, K66, K55.21, K92, I31.2, R58, R31.0, R04.0, M25.019
Obesity	278	E66.9
Pulmonary fibrosis	516.31	J84.10

*ICD-9 and 10* International Classification of Disease ninth and tenth edition, *COPD* chronic obstructive pulmonary disease

.

## Appendix 4

See Table 6

**Table 6:** Median (IQR) hospital length of stay among patients with low risk venous thromboembolism (VTE) complications who were admitted for hospital management by type of VTE, during the study period (N = 7,293)

Year	DVT	PE	Total	*p*-value
2013	4.7 (2.7–7.7)	4.0 (2.2–6.5)	4.5 (2.5–7.1)	< 0.01
2014	4.4 (2.7–7.9)	3.4 (2.1–6.0)	4.0 (2.4–7.0)	< 0.01
2015	4.9 (2.9–8.0)	3.2 (2.0–6.2)	4.1 (2.4–7.5)	< 0.01
2016	5.0 (2.8–9.1)	3.1 (1.8–5.6)	4.1 (2.1–7.8)	< 0.01
2017	5.0 (2.4–9.2)	3.1 (1.9–5.7)	4.0 (2.1–7.9)	< 0.01
2018	4.9 (2.4–9.0)	2.9 (1.8–5.3)	3.9 (2.0–7.7)	< 0.01

*VTE* venous thromboembolism, *IQR* interquartile range

.
